# Recurrence of suicidal ideation due to treatment with antidepressants in anxiety disorder: a case report

**DOI:** 10.1186/1752-1947-1-166

**Published:** 2007-12-03

**Authors:** Doron Todder, Bernhard T Baune

**Affiliations:** 1 Department of Psychiatry, Ben-Gurion University, Beer-Sheva, Israel; 2 Department of Psychiatry, University of Muenster, Germany; 3 Department of Psychiatry, James Cook University, Australia

## Abstract

This report describes a patient suffering from panic disorder who developed repeated suicidal ideation specifically due to the treatment with Venlafaxine. A first suicide attempt years ago occurred while being treated with Venlafaxine. Subsequent treatment with SSRIs or other antidepressants involved no suicidal ideation. Re-commencement of Venlafaxine four years later immediately led to a second suicide attempt. This unwanted effect subsided immediately after switching to another SNRI (i.e. Duloxetine). The case report underlines the importance of onset of suicide risk in panic disorders due to specific antidepressants.

## Introduction

Since the introduction of the first specific serotonin reuptake inhibitors (SSRI) Fluoxetine, a concern emerged regarding the risk of developing suicide ideation as a rare side effect [1]. During 2003, Britain's Committee on Safety of Medicines issued a warning about the dangers of developing suicide as side effect, first for Paroxetine and then extended the warning to all the new antidepressants [2]. The USA Food and Drug Administration followed, embracing the warning for children, and then extended the warning to adults [3].

Most of the case reports that were published on the subject dealt with depressive patients. Some of these case reports described treatment-resistant patients or patients who were treated with higher than common-practice drug doses. All these drawbacks limited the ability to reach conclusive answers regarding the true connection between suicide and antidepressant treatment [4].

The following description of a patient is unique because he suffered from anxiety disorder and was neither depressed nor experiencing suicide ideation prior to the beginning of the antidepressant treatment. This case highlights the idiosyncratic response of suicidal ideation emerging from the treatment with antidepressants.

## Case presentation

We report on a 19 year-old-male patient who suffers from panic disorder according to the DSM-IV (Diagnostic and Statistical Manual of Mental Disorders, version IV) [5] criteria since the age of 15. He was also diagnosed with co-morbid narcissistic personality disorder, but without any drug or alcohol abuse in the past. No medical or other health problems were known or present. Onset of panic attacks was at age 15 when his parents divorced. Initial pharmacological treatment with Fluoxetine 20 mg (titered up within a few weeks) was combined with individual and family psychotherapy on an outpatient level. Due to lack of response the patient was switch to Fluvoxamine 150 mg after 2 months. Despite this intensified treatment regimen, panic attacks continued and his overall state deteriorated eventually leading to hospitalization to a youth inpatient ward. Treatment was changed to Venlafaxine initially with 75 mg/day in the morning. Almost immediately the patient experienced frequent suicide thoughts along with feelings of despair and hopelessness. The thoughts frightened him to the point of suffering frequent panic attacks. This deterioration was interpreted as a sign of treatment resistance and therefore, Venlafaxine was titered up to 300 mg/day. Subsequently, the patient made his first suicide attempt by drinking acid that was left by a careless hospital worker. No psychosocial factors or stressful life events were detected to have contributed to the suicide attempt.

Following this event, Paroxetine was trialed up to 20 mg/d leading to almost full remission and only rare panic attacks during the following 4 weeks. At that point in time, when the patient was discharged home, his general functioning was normal. In the next 4 years the patient continued on Paroxetine 20 mg/d because when he tried lowering this dose according to his psychiatrist, he felt more anxious. He was regularly followed-up during this time and experienced rare classic panic attacks mostly during stressful times.

After 4 years of continuous antidepressant treatment with Paroxetine, the patient started to complain about delayed ejaculation which was attributed to Paroxetine. As a consequence other antidepressants were trialed, none of which improved his sexual disturbance or caused any worsening of his anxiety. Finally when Venlafaxine was trialed again due to lack of effective alternatives with no/little side-effects, the patient complained about frequent suicide thoughts after one week of Venlafaxine XR 75 mg. Other medication was not used at that time. He appeared in the outpatient clinic as very anxious, reported being afraid to stay on his own, was terrified that he might lose control and to unintentionally harm himself. The suicidal thoughts were experienced in the same way as the suicidal ideation and suicide attempt from his adolescent years. After switching to Duloxetine 60 mg/day, the suicidal thoughts completely disappeared.

## Discussion

In the last years, several large retrospective analyses were carried out in order to expand the understanding of the phenomenon of suicidal ideation due to SSRI medication. These studies pointed out that regarding the risk of developing suicide acts, the new antidepressants present with a similar risk of suicidal ideation as the old TCA, [6,7]. It was also concluded that antidepressants as a group elevate the risk for suicide compared to placebo [8].

On the other hand, many studies confirm the reciprocal connection between the increased use of antidepressants and the decline of suicide. For example, in a study conducted in Scandinavia, victims of suicide were compared to people who died as a result of natural or traumatic events. The suicide group was relatively undertreated with antidepressants [9]. A more recent report applying aggregated data suggests that the increased use of SSRIs is related to decreased suicide rates whereas TCA prescriptions were related to increased suicide rates [10]. On a similar point, a retrospective analyses of the relationship between prescriptions of SSRIs and suicide rates suggests that the decrease of SSRI prescriptions for children and adolescents, both in the United States and the Netherlands, after U.S. and European regulatory agencies issued warnings about a possible suicide risk with antidepressant use in pediatric patients, was associated with increases in suicide rates in children and adolescents [11].

Combining this research the conclusion is that for the society as a group, the use of antidepressants is beneficial. Nevertheless, for certain individuals, these medicines could cause suicide ideation and put them in great risk [12]. Therefore, identifying these patients is important from a clinical and legal perspective.

The raising awareness of the suicidal risk for developing suicidal ideation and behavior during treatment with antidepressants causes a dramatic change of the attitude toward these drugs. The presented case demonstrates for the first time a recurrence of suicidality following antidepressants in patients with anxiety disorder. Therefore, suicidality as a direct consequence of antidepressant treatment is not restricted to patients with major depressive disorder. Improved understanding of the risks and detection of early clinical warning signs while using antidepressants is therefore vital for these patients.

The understanding of the precise mechanisms by which antidepressants may cause suicidality is still lacking. Few theories were put forward along the years that can be divided into two groups: either attributed to depressive symptoms or attributed to the drug itself. Theories about attribution to depressive symptoms claimed that this phenomenon is merely a consequence of a clinical worsening of the specific disorder which is known as the "paradoxical suicide" [12,13]. Others speculated that these patients may suffer from an undiagnosed bipolar disorder [14]. Theories that focus on side effect of the drug claimed that the development of akathisia due to antidepressant treatment possibly is the cause for suicidality [15]. Beyond these explanations, our case contributes to other theories as follows. Idiosyncratic response to psychotropic agents might have occurred in both susceptible individuals as suicidality subsided when switching to another drug of the same class. This theory gains more strength when considering that even treatment of normal subjects could result in suicide ideation [4]. Furthermore, a sudden onset of suicidal feelings is a well known but still relatively underestimated and not widely understood phenomenon. Patients often describe feelings of hopelessness and despair that may develop after starting treatment with an antidepressant [12] that may lead to suicidality. The classical action of the reserpin can serve as a model for such action. In addition, auto-aggressive feelings are possibly part of panic attacks as reported by George et al. who described three patients developing auto-aggressive and suicidal thought during panic attacks [16]. The authors described the suicidal ideation as sudden "attacks" resembling panic attacks.

Hypothetically, biological alterations in the serotonergic system might have contributed to the suicidal ideation/attempt in the presence of an SSRI whereas this effect ceased with the pure NSRI was used. The following mechanism for a reduced serotonergic activity induced by SSRI can be suggested as an explanation of our case observation. It is reported that the increase of the concentration of 5-HT in the extracellular brain space through most antidepressants by preventing its reuptake is offset by a negative feedback operating at the 5-HT cell-body level [17]. It was shown that the inhibition of 5-HT reuptake produced by administration of SSRIs can cause a marked enhancement of the extracellular concentration of 5-HT in the midbrain raphe nuclei [18-20] and accounted for the suppression of 5-HT cell firing [21,22]. Consequently, the presence of an SSRI can lead to a reduced activity of serotonin-mediated neuronal activity possible related to abnormal behaviour such as suicidal ideation. Unfortunately, since it was not possible to clarify the biological/serotonergic make-up of our case, these assumptions require further investigations.

## Conclusion

Nearly all research on suicide rates that show biological [23], psychological [14,24] and social [25] factors that contribute to suicide risk, do not differentiate explicitly between risk for depression and anxiety disorders. More specifically, little attention has been given to and no clinical experience reported, if modern antidepressants do contribute to suicide risk in anxiety disorders [4]. More clarity on the diagnosis specific suicide risk appears to be important for clinical practice and also for the understanding of the underlying mechanisms of suicide risk due to antidepressants. An enhanced understanding of the anxiety specific suicide risk might help to improve clinical practice to anticipate and early identify individuals developing suicidal ideation while treated with antidepressants.

## Conflict of interests

The author(s) declare that they have no competing interests.

## Authors' contributions

DT was the treating psychiatrist of the patient. Both DT and BB drafted the manuscript, read and approved the final manuscript.

## Consent

Written informed consent was obtained from the patient for publication of this case report and any accompanying images. A copy of the written consent is available for review by the Editor-in-Chief of this journal.
